# Benefits in Oral Health during Orthodontic Treatment of Patients Aged 17 to 21 Years

**DOI:** 10.1055/s-0042-1744374

**Published:** 2022-09-12

**Authors:** Çeljana Toti, Etleva Droboniku, Gerta Kaçani, Michele Tepedino, Aida Meto, Luca Fiorillo, Cesare D'Amico, Dorjan Hysi, Kevin Ndreu, Marco Cicciù, Olja Tanellari

**Affiliations:** 1 Department of Orthodontics, Faculty of Dental Medicine, University of Medicine, Tirana, Albania; 2 Department of Dental Therapy, Faculty of Dental Medicine, University of Medicine, Tirana, Albania; 3 Department of Biotechnological and Applied Clinical Sciences, University of L'Aquila, L'Aquila, Italy; 4 Department of Dentistry, Faculty of Dental Medicine, University of Western Balkans, Tirana, Albania; 5 Department of Dentistry, Faculty of Dental Sciences, University of Aldent, Tirana, Albania; 6 Department of Biomedical and Dental Sciences, Morphological and Functional Images, University of Messina, Messina, Italy; 7 Multidisciplinary Department of Medical-Surgical and Odontostomatological Specialties, University of Campania “Luigi Vanvitelli,” Naples, Italy; 8 Private Dental Clinic, Tirana, Albania

**Keywords:** oral health-related quality of life, orthodontic treatment, oral health impact profile

## Abstract

**Objectives** The aim of the present study was to evaluate the effects of orthodontic treatment on patients' oral health-related quality of life (OHRQoL) in a population aged 17 to 21 years. The influence of gender and malocclusion severity was also investigated.

**Materials and Methods** In the present study, 108 patients were enrolled. Each patient completed a questionnaire about oral health impact profile (OHIP)-14 before treatment and after 12 months of treatment. The severity of the initial malocclusion was evaluated through the index for orthodontic treatment need (IOTN).

**Statistical Analysis** Statistical analysis was performed to assess the presence of difference in OHRQoL score before and after the treatment, and the influence of gender and IOTN score on the observed outcomes.

**Results** We found no significant differences related to gender regarding their perception of how malocclusion affects the quality of life before orthodontic treatment. Moreover, no significant differences were found between males and females regarding their perception of how orthodontic treatment affects the quality of life 12 months after orthodontic treatment. Also, the analysis showed no statistically significant difference between males and females in the correlation IOTN-OHIP for Grades 2 and 3. A statistically significant difference between males and females was found only in Grade 4, both before ( *p* = 0.046) and after treatment ( *p* = 0.051).

**Conclusion** Finally, OHIP-14 can be a valuable instrument to assess the perceptions and the expectations of patients toward orthodontic treatment.

## Introduction

Oral health-related quality of life (OHRQoL) is an integral part of overall health and well-being recognized by the World Health Organization (WHO) and the Global Oral Health Program. Many authors define OHRQoL as a tool that “reflects the comfort of people when they eat, sleep, engage in social interactions, in their self-esteem, in satisfaction about their oral health”. 1 2 Other authors consider it as a result of the interaction between oral health conditions, social factors, and the rest of the body. 3 4 The effects of oral health and diseases related to it, teeth's appearance, malocclusion, and treatment of such anomalies on emotional, mental, and social health of patients, have been the focus of attention of clinicians and researchers all over the world during the last decades. Malocclusion is a condition that affects the quality of life.

Studies in adolescents have confirmed the impact of malocclusion on their quality of life. 5 6 7 A meta-analysis found that patients undergoing treatment for malocclusion had significantly lower scores of OHRQoL compared with individuals without malocclusion. 8 Consistent improvement in social well-being and the quality of life has been generally documented after the first 12 months of orthodontic treatment in Chinese adolescents. 9 OHRQoL is important not only for the patient's well-being but also for motivation and compliance, especially during the first stage of treatment. 10

A recent study 11 concluded that the quality of life in adults is much more sensitive to malocclusion compared with adolescents and that the social and emotional aspects are particularly affected. Environmental, social, cultural, and economic factors have an important influence on the perception of OHRQoL, as demonstrated by a systematic review, 12 therefore data from different populations cannot be easily generalized to a different one.

We have very little data or limited evidence regarding this issue in the Albanian population. 13 For this reason, we performed a study with the aim to determine benefits during orthodontic treatment in terms of OHRQoL in Albanian young adults. The influence of gender and of the initial Index of Orthodontic Treatment Need (IOTN) score was also investigated.

## Materials and Methods

The present study was performed from May 2019 to September 2020. The patients provided their consent after being informed at the beginning of the procedures. Approval of the present study was obtained from the Local Ethics Committee (Protocol no. 362/2019).

### Patients' Selection and Sample Size

The participation was voluntary and all patients had to meet the following criteria to be included in the study: (i) aged 17–21 years old; (ii) Albanian citizenship; (iii) not suffering from endocrine or chronic diseases; (iv) not having severe craniofacial disorders; and (v) no previously undergoing any orthodontic treatment. Those patients who had a highly compromised periodontal status were excluded. The sample size was determined using the Power and Sample Size Calculation program (PS, version 3.0, Nashville, TN, USA). Based on similar studies, a sample of 81 individuals would provide a statistical power of 90% to identify a 5% significant difference in OHRQoL between the time before treatment and 12 months after orthodontic treatment start. During the initial screening, 112 patients were considered, but only 108 of them were recruited in the study. In particular, two patients moved to live abroad during the study, and the other two delayed their appointment and were evaluated after the defined period of 12 months. Nevertheless, because those four patients represented a 3.5% drop-out rate, the possible attrition bias derived was considered negligible.

### Oral Health Impact Profile (OHIP-14) Survey

Oral health impact profile (OHIP-14) was considered an appropriate survey to use in this study. It consists of 14 questions; it is simple and does not take much time to complete ( Table 1 ). Responses are coded 0 for the answer “Never,” and 1 for the answers “Hardly ever,” “Occasionally,” “Often,” and “Very often.” We had trained six assistants to help the patients to complete the survey. The questionnaire was administered to the patients right after the clinical examination and the IOTN assessment, but before explaining the therapeutic plan, to avoid that their perception would be affected by the information regarding the orthodontic treatment plan. Patients completed their survey independently: they were assured that the information was confidential, they were advised to recall their last year's experiences, and to answer the survey honestly after reading the questions carefully, not consulting with others while answering the questions.

**Table 1:** DHC uses a 5-level scale depending on the degree of malocclusion

Degree of malocclusion according to DHC	Clinical findings
Grade I	No need for orthodontic treatment (variations in occlusion are quite small including displacements less than 1 mm)
Grade II	Minimal need for treatment (overjet greater than 3.5 mm but less than 6 mm with labial competence at rest; reverse overjet greater than 0 mm but less than or equal to 1 mm; overbite greater than 3.5 mm without gingival contact; anterior or posterior crossbite with less than or 1 mm displacement of ICP and CP position; open lateral or anterior bite greater than 1 mm but less than or equal to 2 mm; displacement of teeth greater than 1 mm but less than or equal to 2 mm)
Grade III	Moderate need for treatment (overjet greater than 3.5 mm but less than or equal to 6 mm with labial incompetence at rest; reverse overjet greater than 1 mm but less than or equal to 3.5 mm; overbite with gingival contact but no signs of trauma; anterior or posterior crossbite with less than or 1–2 mm displacement of ICP and CP position; open lateral or anterior bite greater than 2 mm but less than or equal to 4 mm; tooth displacement greater than 2 mm but less than or equal to 4 mm)
Grade IV	The essential need for treatment (overjet greater than 6 mm but less than or equal to 9 mm; reverse overjet greater than 3.5 mm but without masticatory or speech problems; reverse overjet greater than 1 mm and less than or equal to 3.5 mm, but with masticatory problems or difficulty speaking; anterior or posterior crossbite with more than 2 mm displacement ICP and CP position; displacement of teeth greater than 4 mm; deep traumatic bite)
Grade V	Extreme need for treatment (defects such as cleft lip or palate; incision larger than 9 mm; reverse incision greater than 3.5 mm and masticatory problems or difficulty speaking; obstructed tooth eruption (with the exclusion of third molars) due to dental cavities, presence of supra-numerary teeth, retained teeth, and other pathological causes; extensive hypodontia with prosthetic problems (absence of more than one tooth in each quadrant, and need for pre-prosthetic treatment)

The same survey was administered to the patients 12 months after the start of the orthodontic therapy. Patients were at different stages of the therapy progress. IOTN is a clinical index that has long been used as an assessment tool in epidemiological studies. 14 The index incorporates both the Dental Health Component (DHC) and the Aesthetic Component (AC). The DHC represents the biological or anatomical aspect of IOTN that records the need for treatment on dental health and functional grounds. AC measures aesthetic impairment and justifies treatment on social-psychological grounds. Thus, it ranks malocclusion in terms of the significance of various occlusal traits for the person's dental health and perceived aesthetic impairment with the intention of identifying those persons who would be most likely to benefit from orthodontic treatment. 15

The dental component is widely used in epidemiological studies. In the present study, the malocclusion severity was determined with the DHC ( Table 1 ) to find a possible correlation between patients' self-assessment of their malocclusion on the one hand, and the degree of malocclusion determined by the doctor on the other.

### Statistical Analysis

All collected data were transferred to an excel sheet and then exported to SPSS version 20.0, to perform statistical analysis. For all categorical variables (including binary/dichotomous and ordinal scale), absolute numbers and corresponding percentages were calculated. For all numerical variables, when the data were subjected to the normal distribution, the corresponding arithmetic means ± standard deviations (SD) were calculated. An independent samples *t* -test was used to assess the differences between groups and between the two-time points. Differences between groups for discrete variables were evaluated with Chi-square and Wilcoxon test. The relationships between the variables were analyzed through Kendal's tau correlation coefficients. A two-way analysis of variance (ANOVA) was used to compare the OHIP scores between gender and different IOTN categories. The values of *p* ≤ 0.05 were considered significant.

## Results

### Perception of Malocclusion according to the Gender

The study included 108 individuals. The mean age of the sample was 18.76 ± 2.2 years ( Table 2 ). Comparisons between the mean values of OHIP, before treatment and 12 months after treatment start, showed that there was no statistically significant difference in mean OHIP values for males and females, either in the pre-assessment or in the after 12-month assessment. The preliminary study of the data showed that both males and females had almost the same perception of their malocclusion ( *p* = 0.876) and that they had benefited the same from the orthodontic treatment ( *p* = 0.634) ( Table 3 ).

**Table 2:** Composition of sample by gender

Gender	Cases	Percentage (%)
Male	32	29.6
Female	76	70.4
Total	108	100.0

**Table 3:** Average values for age, IOTN and OHIP

Variables	Total	Gender		*p* -Value
		M ( *n* = 32)	F ( *n* = 76)	
Age	18.76 ± 2.20	18.44 ± 1.95	18.91 ± 2.30	0.314
IOTN	2.64 ± 0.71	2.56 ± 0.76	2.67 ± 0.70	0.475
OHIP: before	11.73 ± 3.22	11.66 ± 3.15	11.76 ± 3.28	0.876
OHIP: after 12 months	6.64 ± 3.84	6.38 ± 3.26	6.76 ± 4.08	0.634

* Student's *t* -test.

The Student's *t* -test showed no statistically significant difference among females and males regarding the age and IOTN ( Table 3 ).

### Comparing Assessments Before and After Twelve Months for Each Questionnaire's Item

There was a statistically significant difference between the OHIP scores before and after 12 months into treatment, regarding all the survey's questions ( Tables 4 and 5 ). Significant differences were found for response “Never” in the Q.5 (5 patients before treatment, versus 44 patients after 12 months) and in the Q.6 (9 patients before treatment versus 51 patients after 12 months). Significant differences were also found for the response “Very often” in the Q.6 (41 patients before treatment versus 1 patient after 12 months). For the Q.7 & 8, the differences for the response “Never” were significant, but not as obvious as in the Q.5 & 6. Also in the Q.9, significant differences were found for the “Never” response (13 patients before treatment versus 61 patients after 12 months) and the “Very often” response (20 patients before treatment versus no patient after 12 months). Significant differences for the “Never” response were found for the Q.10 (8 patients before treatment versus 50 of them 12 months after) and 11 (13 patients before treatment versus 66 patients after 12 months).

**Table 4:** Evaluation of OHIP score before treatment

Questions	Never	Almost never	Occasionally	Often	Very often
1	33 (30.6)	39 (36.1)	31 (28.7)	5 (4.6)	–
2	30 (27.8)	56 (51.9)	20 (18.5)	2 (1.9)	–
3	10 (9.3)	37 (34.3)	48 (44.4)	13 (12.0)	–
4	18 (16.7)	42 (38.9)	37 (34.3)	10 (9.3)	1 (0.9)
5	5 (4.6)	11 (10.2)	18 (16.7)	35 (32.4)	39 (36.1)
6	9 (8.3)	11 (10.2)	18 (16.7)	29 (26.9)	41 (38.0)
7	24 (22.2)	48 (44.4)	28 (25.9)	7 (6.5)	1 (0.9)
8	27 (25.0)	51 (47.2)	26 (24.1)	4 (3.7)	–
9	13 (12.0)	14 (13.0)	26 (24.1)	35 (32.4)	20 (18.5)
10	8 (7.4)	6 (5.6)	19 (17.6)	45 (41.7)	30 (27.8)
11	13 (12.0)	13 (12.0)	13 (12.0)	40 (37.0)	29 (26.9)
12	19 (17.6)	10 (9.3)	21 (19.4)	35 (32.4)	23 (21.3)
13	15 (13.9)	9 (8.3)	19 (17.6)	25 (23.1)	40 (37.0)
14	21 (19.4)	11 (10.2)	11 (10.2)	11 (10.2)	47 (43.5)

**Table 5:** Evaluation of OHIP score after twelve months of treatment

Questions	Never	Almost never	Sometimes	Often	Very often
1	40 (37.0)	47 (43.5)	19 (17.6)	1 (0.9)	–
2	44 (40.7)	46 (42.6)	17 (15.7)	1 (0.9)	–
3	34 (31.5)	28 (25.9)	42 (38.9)	3 (2.8)	1 (0.9)
4	46 (42.6)	35 (32.4)	26 (24.1)	1 (0.9)	–
5	44 (40.7)	27 (25.0)	20 (18.5)	10 (9.3)	7 (6.5)
6	51 (47.2)	27 (25.0)	17 (15.7)	12 (11.1)	1 (0.9)
7	63 (58.3)	32 (29.6)	10 (9.3)	3 (2.8)	–
8	58 (53.7)	37 (34.3)	(10.2)	2 (1. 9)	–
9	61 (56.5)	31 (28.7)	10 (9.3)	6 (5.6)	–
10	50 (46.3)	29 (26.9)	17 (15.7)	10 (9.3)	2 (1. 9)
11	66 (61.1)	27 (25.0)	8 (7.4)	4 (3.7)	3 (2.8)
12	64 (59.3)	27 (25.0)	12 (11.1)	4 (3.7)	1 (0.9)
13	69 (63.9)	28 (25.9)	4 (3.7)	4 (3.7)	3 (2.8)
14	79 (73.1)	14 (13.0)	10 (9.3)	3 (2.8)	2 (1. 9)

It was also observed that before treatment, the highest percentages of answers were for the options “Occasionally,” “Often,” and “Very often.” Table 6 shows a comparison of assessment before and after 12 months only for the options “Occasionally,” “Often” and “Very often,” considering those categories as “problematic-group.” The results for the options “Never” and “Hardly Ever” are excluded.

**Table 6:** Comparison of assessment before treatment and after twelve months into treatment for answers. “Occasionally,” “Often,” “Very often”

Questions	Before (occasionally, often, very often)	After (occasionally, often, very often)	*p* -Value
1	36 (33.33)	20 (18.52)	ns
2	22 (20.37)	18 (16.67)	ns
3	61 (56.48)	46 (42.59)	ns
4	48 (44.44)	27 (25.00)	ns
5	92 (85.19)	37 (34.26)	0.037
6	88 (81.48)	30 (27.78)	0.003
7	36 (33.33)	13 (12.05)	ns
8	30 (27.78)	13 (12.05)	ns
9	81 (75.00)	16 (14.81)	<0.001
10	94 (87.04)	29 (26.85)	0.003
11	82 (75.93)	15 (13.89)	<0.001
12	79 (73.15)	16 (14.81)	<0.001
13	83 (76.85)	15 (13.89)	<0.001
14	76 (70.37)	15 (13.89)	<0.001

Abbreviation: ns, non-significant.

Significant differences were found for domains “Psychological discomfort” (Q.5 & Q.6), “Psychological disability” (Q.9 & Q.10), “Social disability” (Q.11 & Q.12) and “Handicap” Q.13 & Q.14). It is clearly shown the decline in the number of patients being self-conscious (Q.5) ( *p* = 0.037), feeling tense (Q.6) ( *p* = 0.003), finding difficult to relax (Q.9) ( *p* < 0.001), being embarrassed (Q.10) ( *p* = 0.003), being irritable (Q.11) ( *p* < 0.001), having difficulty doing usual jobs (Q.12) ( *p* < 0.001), feeling that life in general was less satisfying (Q.13) ( *p* < 0.001), being totally unable to function (Q.14) ( *p* < 0.001) because of problems with their teeth, mouth, or denture. Differences were also observed for domains “Functional,” “Physical,” and “Physical disability” (Q. 1, 2, 3, 4, 7 and 8), but these differences were not significant.

### Correlation between IOTN and Assessments in the Survey

**Fig. 1:**
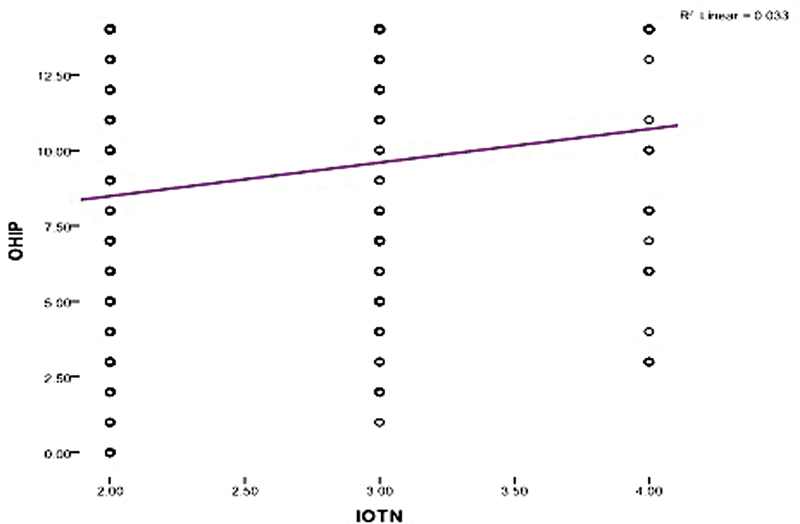
The correlation between OHIP and IOTN.

Kendal's tau correlation coefficient was used to estimate the correlation between OHIP and IOTN ( Fig. 1 ). It was observed that there was a statistically significant positive correlation between OHIP and IOTN ( *r* = 0.182, *p* = 0.006).

Student's *t* -test was used to compare OHIP mean values before treatment and 12 months after treatment start with regard to the degree of malocclusion for males ( Table 7 ) and females ( Table 8 ). Statistically significant differences were found in OHIP mean values at both time intervals for all degrees of malocclusion for males.

**Table 7:** Comparison of average scores of OHIP by the grades of IOTN in male patients before and after twelve months of orthodontic treatment

Male patients			
IOTN	OHIP before	OHIP twelve months after	*p* -Value*
Grade 2	11.21 ± 3.19	5.53 ± 2.76	< 0.001
Grade 3	11.38 ± 3.19	6.25 ± 2.92	< 0.001
Grade 4	11.38 ± 3.70	9.80 ± 3.90	< 0.001

* Student's *t* -test.

**Table 8:** Comparison of average scores of OHIP by the grades of IOTN in female patients before and after twelve months of orthodontic treatment

Female patients			
IOTN	OHIP before	OHIP twelve months after	*p* -Value*
Grade 2	11.29 ± 3.54	5.97 ± 4.20	< 0.001
Grade 3	12.26 ± 3.08	6.94 ± 3.50	< 0.001
Grade 4	11.90 ± 3.00	9.00 ± 4.81	0.027

* Student's *t* -test.

## Discussion

The results of the present study revealed that the malocclusion affected the patients' relationships and their daily activities, making life less satisfactory. These conclusions are also consistent with data from the literature. 16 17 18 19 20

The patients were in general satisfied with their orthodontic treatment. Moreover, the correction of the malocclusion made it easier for these patients to cope with bullying or frustrating situations. These results are consistent with those of other studies. 4 21 It is interesting to observe such an improvement in OHRQoL even during the initial stage of treatment (i.e., the first 12 months): this finding implies that a partial improvement in the malocclusion is sufficient to determine a significant positive effect on patient's OHRQoL, and that it is not necessary to reach the end of treatment for it. The implications of this phenomenon are related to the possible effects on compliance and motivation. Although this was not assessed in the present study, a patient who perceives his OHRQoL improving as the treatment progresses will probably be more cooperative and willing to continue his treatment. This particular aspect will deserve further investigation in the future.

Considering the need for treatment as indicated by IOTN-DHC, there were significant differences in OHRQoL between treatment needs levels although no gradient consistency was evident.

These findings are consistent with an earlier study from Brazil, 22 though they are in contrast to other studies 18 19 conducted where the impact on OHRQoL has followed a more stable pattern depending on the degree of malocclusion.

Other systematic reviews 20 23 included studies that were conducted for children aged 12 to 15 years, supporting a link between the high severity of malocclusion and increased impact. The correlation between malocclusion and the need for orthodontic treatment has been demonstrated in other studies. 24 25 26 27 Other studies showed a moderate association between malocclusion/the need for orthodontic treatment and OHRQoL in adults, adolescents, and children. 28 29

One of the limitations of this study is the lack of a control group, but each subject in this study acted as control of him/herself. The study showed the differences before treatment and after 12 months, not the difference with the end of treatment. A longer period of observation may influence the results of our findings. However, the results of this study provide a baseline for future studies.

## Conclusions

There were no significant differences related to gender regarding the perception of how malocclusion affects the quality of life before and 12 months after orthodontic treatment starts. There were statistically significant differences between the mean of OHIP before orthodontic treatment and 12 months after the orthodontic treatment start, mostly observed in the improvement of psychological comfort, physical ability, and social well-being. There was a correlation between OHIP and the degree of malocclusion.
